# Genetic identification of eggs from four species of Ophichthidae and Congridae (Anguilliformes) in the northern East China Sea

**DOI:** 10.1371/journal.pone.0195382

**Published:** 2018-04-05

**Authors:** Hae-young Choi, Jina Oh, Sung Kim

**Affiliations:** 1 Marine Ecosystem and Biological Research Center, Korea Institute of Ocean Science & Technology, Yeongdo-gu, Busan Metropolitan City, Korea; 2 Marine Biology, Korea University of Science & Technology, Daejeon, Korea; Shanghai Ocean University, CHINA

## Abstract

We report the first genetic identification of eggs of four species of Anguilliformes caught in the northern East China Sea during August 2016, where leptocephali and adults have been collected. The species were *Ophisurus macrorhynchos* and *Echelus uropterus* belonging to the Ophichthidae, and *Ariosoma majus* and *Gnathophis heterognathos* belonging to the Congridae. The eggs were identified using three molecular genetic markers (mitochondrial 12S rRNA, 16S rRNA, and cytochrome c oxidase subunit 1), sequences obtained from local adult specimens, and geographical distribution data. All eggs were in the early or middle developmental stages. For all species except *A*. *majus*, the eggs were found near the range of small leptocephali in the East China Sea and the southern Korean Peninsula, which indicates these species had spawned along the continental near these areas during the summer.

## Introduction

Members of the Anguilliformes have dramatic life histories, and Ophichthidae and Congridae live in shallow water with most spawning areas appearing to be over or along the edge of the continental shelf [1]. The larvae of Anguilliformes, called leptocephali, are transparent and laterally compressed with larval durations for months to years [2]. Analysis of newly hatched preleptocephali and small leptocephali increases the accuracy of locating spawning areas [3–5]. Small leptocephali of the Ophichthidae and Congridae are found near the continental shelf and edge of the shelf break in the East China Sea and over the continental shelf of the southern Korean Peninsula [6,7]. However, the presence of eggs, representing spawning events, has not yet been reported in these areas.

The discovery of eggs would provide crucial evidence supporting this region as a spawning area [8]. One of the biggest advantages of using eggs to search spawning areas is that eggs in the early developmental stage are found nearer to the area where spawning activity occurs than larvae [9,10]. Hatching times of artificially fertilized Anguilliformes eggs are within a few days of spawning: 1.5 days for *Ariosoma shiroanago major* (valid as *A*. *majus*) at 25.2–26.4°C and 2.5 days for *Gnathophis nystromi nystromi* (valid as *G*. *heterognathos*) at 24.0–26.3°C [11]. On the other hand, 17.5-day-old leptocephali of *Anguilla japonica* have been found about 260 km from the location where eggs and newly hatched preleptocephali appeared [12,13].

It is fundamental to accurately identify the species of any such eggs. Anguilliform eggs have a large diameter and wide perivitelline space compared to eggs of other taxa [14,15]. They are difficult to identify morphologically to the species level because their morphological features are similar among species [16]. This difficulty can be overcome through DNA barcoding [8,15–19]. Representative molecular genetic markers for DNA barcoding include mitochondrial DNA (12S and 16S rRNA, and COI). Comparison of genetic markers of eggs with those of adults that have been classified at the species level makes it possible to identify species of the fish eggs [20].

Around the continental shelf of the East China Sea and the southern Korean Peninsula, where small leptocephali of Ophichthidae and Congridae are found, could be a spawning area for these families. We collected eggs from the northwestern Pacific, including areas where leptocephali appear, and identified four species of Ophichthidae and Congridae using molecular genetic markers. We compared the distributions of eggs collected in this study and leptocephali of the same species from the literature.

## Materials and methods

### Egg collection

**Fig 1:**
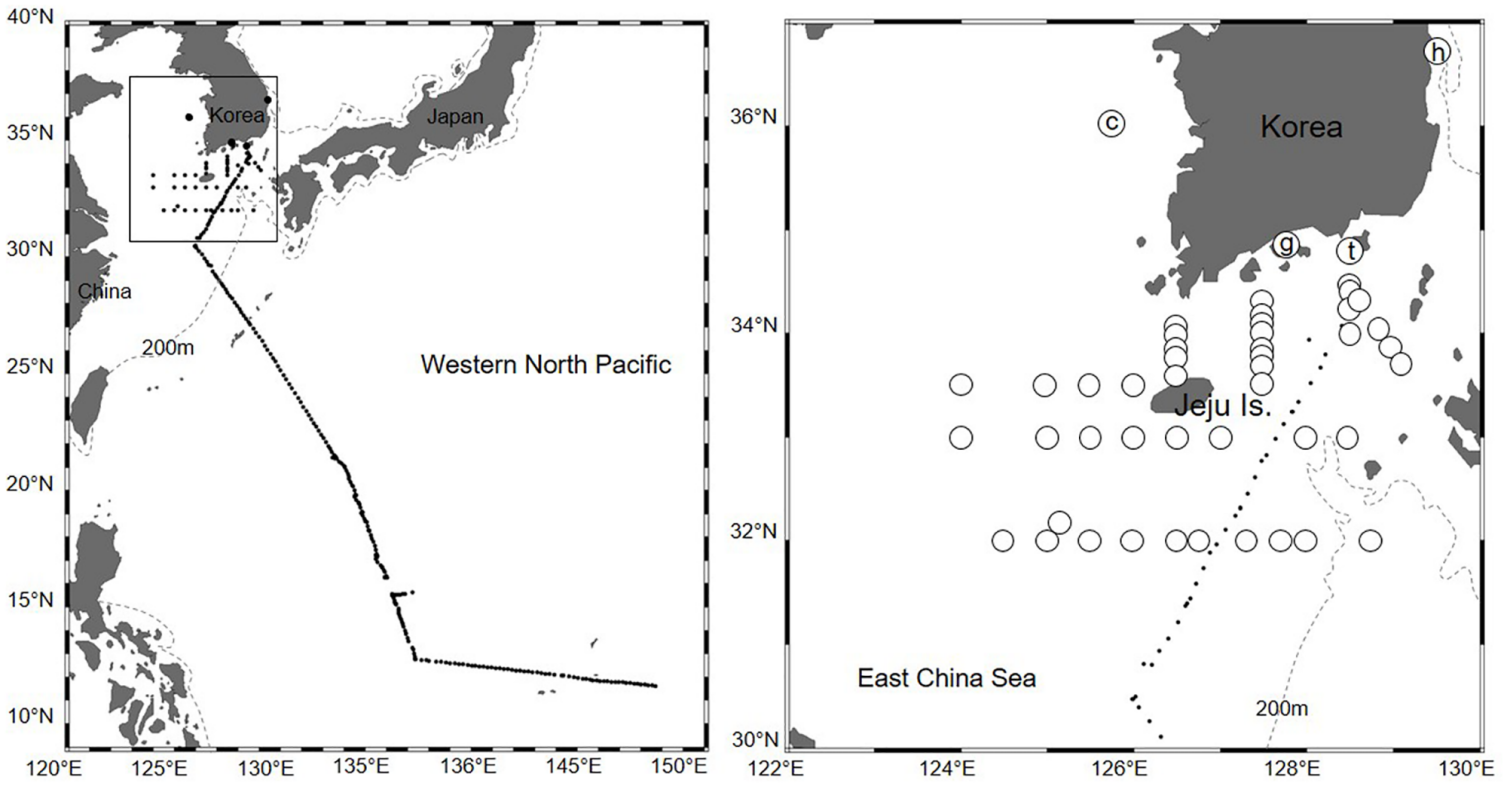
Sampling stations of marine teleost eggs in 2009 and 2012–2016 in this study. The area within the rectangle in the left map is enlarged on the right. Circled letter c, g, h, and t, open circle, and black dot indicate the sampling stations; the details about each sampling area are listed in Table 1. Dashed line means 200m depth contour.

Marine teleost eggs were collected near the Korean Peninsula and the northern East China Sea in 2009 and 2013–2016 and in the northwestern Pacific in 2012 (Fig 1). Fish eggs were sampled using zooplankton nets (diameter 60–80 cm; mesh 220–300 μm; towing time 5–7 min) near Ochung Island (July 2009), Hupo, and Gwangyang Bay (July and August in 2013 and 2014), Korea and the northern East China Sea (during research cruises on the R/Vs *Onnuri* and *Eardo* in June and November 2015, April and August 2016). Fish eggs were also collected using an underwater pump (pumping depth 3 m; mesh 300 μm; flux ~10 m^3^/h) from the Tongyoung Marine Science Station of Korea Institute of Ocean Science & Technology (KIOST) (biweekly from May 2013 to December 2015, total 751 h) and in the northwestern Pacific: from Korea, the East China Sea, the Philippine Sea, and to near Guam aboard the R/V *Onnuri* in 2012 (Table 1). All these investigations were approved by the KIOST within the Ministry of Oceans and Fisheries. Fish eggs were sorted under a stereoscopic microscope (Stemi 2000-C, Zeiss, Germany). They were placed in ethanol or a lysis reagent after sorting. Animal welfare protocols were not required, as the Institutional Animal Care and Use Committee require approval only when vertebrate eggs are maintained until hatching. Sea-surface temperatures were measured using a conductivity, temperature, and depth analyzer (SBE 19-plus, Sea Bird Electronics Inc., Bellevue, Washington, USA).

**Table 1:** The sampling areas and methods used to collect marine teleost eggs in this study.

Sampling site	Sampling date	No. of stations	Sampling method	No. of eggs	Species identification^†^	Note^‡^
Off Ochung Is.	22 Jul 2009	6	Zooplankton net*	679	COI	Circled letter c
Off Hupo	6 Aug 2013	10	Zooplankton net**	5410	COI	Circled letter h
	23 Jul 2014	10	Zooplankton net**	2035	COI	
Gwangyang Bay	29 Aug 2013	10	Zooplankton net**	1473	COI	Circled letter g
	25 Jul 2014	10	Zooplankton net**	3782	COI	
Northern East China Sea	1–8 Jun 2015	24	Zooplankton net***	60	COI	Open circle^¶^
	7–14 Nov 2015	17	Zooplankton net***	99	COI	
	26 Apr 2016	5	Zooplankton net***	11	12S, 16S, COI	
	16–24 Aug 2016	28	Zooplankton net***	224	COI	
Western North Pacific	4–20 Jun 2012	326	Underwater pump	577	16S	Black dot
Tongyoung Marine Science Station	May 2013-Dec 2015 biweekly	1	Underwater pump	19082	COI	Circled letter t

* Oblique towing.

** horizontal towing.

*** vertical towing.

^†^ Species identification is based on molecular genetic markers (12S rRNA, 16S rRNA, and COI sequences of mitochondrial DNA).

^‡^ Symbols in the note indicate the sampling stations shown in Fig 1.

^¶^ Stations for each survey are shown in S1 Fig.

### Species identification

Genomic DNA of each egg was extracted using a DNeasy Blood and Tissue Kit (QIAGEN, Hilden, Germany) according to the manufacturer’s protocol. The COI genes or 16S genes (for the survey conducted on 4–20 June 2012 only) were first analyzed (Table 1). Extracted gDNA was subjected to amplification. Tables 2 and 3 show the primer sets used [21–23] and the thermal cycle profiles employed. PCR products were purified using a Universal DNA Purification Kit (TIANGEN, Shanghai, China) and sequenced on a 3730xl DNA Analyzer (Applied Biosystems, Waltham, Massachusetts, USA). The sequences were trimmed and aligned using Geneious 9.1.7 (Biomatters, Auckland, New Zealand). The COI or 16S sequences were compared with all available sequences using the Basic Local Alignment Search Tool [24]. Sixteen eggs collected around the northern East China Sea (16–24 August 2016) were classified by COI as candidate Anguilliformes. PCR was performed for further analysis of 12S and 16S rRNA genes.

**Table 2:** Primers used to amplify three regions on eggs and adult fish.

DNA fragments	Primer name	Sequence (5’-3’)	Reference
12S	12S-F	CAAAGGCCTGGTCCTGACTTTAA	Ji et al. [21]
	12S-R	CCTTCCGGTACACTTACCATGTTA	
16S	16Sar-5’	CGCCTGTTTATCAAAAACAT	Palumbi [22]
	16Sar-3’	CCGGTCTGAACTCAGATCACGT	
COI	VF2_t1	TGTAAAACGACGGCCAGTCAACCAACCACAAAGACATTGGCAC	Ivanova et al. [23]
	FishF2_t1	TGTAAAACGACGGCCAGTCGACTAATCATAAAGATATCGGCAC	
	FishR2_t1	CAGGAAACAGCTATGACACTTCAGGGTGACCGAAGAATCAGAA	
	FR1d_t1	CAGGAAACAGCTATGACACCTCAGGGTGTCCGAARAAYCARAA	

**Table 3:** The PCR conditions employed.

Locus	Condition				
	Initial denaturation	35 cycles			Final extension
		Denaturation	Annealing	Extension	
12S	94°C 5 min.	94°C 30 sec.	56.5°C 30 sec.	72°C 1 min.	72°C 7 min.
16S	94°C 3 min.		56°C 40 sec.	72°C 45 sec.	
COI	94°C 3 min.		52°C 40 sec.	72°C 1 min.	

To enhance the reliability of species identification, sequences from four adult local specimens were also analyzed: *Ariosoma meeki* (Specimen ID, PKU59858), *Gnathophis heterognathos* (referred to previously as *G*. *nystromi nystromi*, PKU54228), *Ophisurus macrorhynchos* (PKU57804) (all obtained from the Marine Fish Resources Bank of Korea), and *Echelus uropterus* (KIP21353) (obtained from the KIOST).

Sequences of three genes (12S rRNA, 16S rRNA, and COI) obtained from the sixteen eggs and the four adult specimens were submitted to the GenBank database (https://www.ncbi.nlm.nih.gov/genbank/), and the accession numbers of each specimen were listed in Table 4.

**Table 4:** GenBank accession numbers of sequences read from fish eggs and adult specimens obtained by this study.

Specimen ID	Species name	GenBank accession number		
		12S	16S	COI
Eggs				
E01	*Ophisurus macrorhynchos*	MF539657	MF539637	MF539677
E02		MF539658	MF539638	MF539678
E03	*Echelus uropterus*	MF539642	MF539622	MF539662
E04		MF539643	MF539623	MF539663
E05		MF539644	MF539624	MF539664
E06		MF539645	MF539625	MF539665
E07	*Ariosoma majus*	MF539640	MF539620	MF539660
E08	*Gnathophis heterognathos*	MF539647	MF539627	MF539667
E09		MF539648	MF539628	MF539668
E10		MF539649	MF539629	MF539669
E11		MF539650	MF539630	MF539670
E12		MF539651	MF539631	MF539671
E13		MF539652	MF539632	MF539672
E14		MF539654	MF539634	MF539674
E15		MF539655	MF539635	MF539675
E16		MF539653	MF539633	MF539673
Adults				
PKU57804	*Ophisurus macrorhynchos*	MF539659	MF539639	MF539679
KIP21353	*Echelus uropterus*	MF539646	MF539626	MF539666
PKU59858	*Ariosoma meeki*	MF539641	MF539621	MF539661
PKU54228	*Gnathophis heterognathos*	MF539656	MF539636	MF539676

Genetic divergence and neighbor-joining trees [25] were analyzed based on 12S, 16S, and COI for the 16 eggs and related taxa, using Kimura two-parameter model [26]. Sequences used in this analysis were from GenBank and BOLD systems and each sequence ID was reported next to the species name on the neighbor-joining tree. Outgroup was the cartilaginous fish *Okamejei kenojei* (NC007173). All analyses were performed using MEGA7 software [27]. All species name was followed by the Catalog of Fishes [28].

### Morphological observations

Some of the sixteen eggs designated as Anguilliformes were photographed onboard the R/V *Onnuri* immediately after collection using a digital camera (EOS 550D, Canon, Japan) attached to a stereoscopic microscope (Stemi 2000-C, Zeiss, Germany). Egg size, oil globules, and the width of the perivitelline space were evident in the photographs.

### Comparison of egg and larval distributions

To compare the distributions of Ophichthidae and Congridae eggs analyzed in this study with those of eggs and leptocephali from literature, we collected geographical information for eggs and larvae of the species: *Ophisurus macrorhynchos*, *Echelus uropterus*, *Ariosoma majus* (known previously as *Ariosoma* sp.7), and *Gnathophis heterognathos*. Details of the surveys from the references [6,7,18,21,29–32] are described in Table 5. The distributions of each species of eggs collected in this study and leptocephali and ocean currents were marked on maps drawn using Ocean Data View [33].

**Table 5:** Marine fish eggs and larvae surveys (including leptocephali) in the western North Pacific from the literature.

Sampling site	Sampling date	No. of stations	Sampling method^†^	Species identification^‡^	Reference
For eggs					
Off Jeju Is.	Aug 2006-Jul 2007	4	1**	cytb	Han et al. [29]
For leptocepahli					
*Ophisurus macrorhynchos*					
Off Jeju Is.	Aug 2013 and 2014	10	2	Morphology & 12S	Ji et al. [7]
*Echelus uropterus*					
East Sea of Korea	Jul-Oct 2010	1	3	Morphology & 12S	Ji et al. [21]
*Ariosoma majus*					
East China Sea	27 Nov-7 Dec 2000	29	4*	Morphology & 16S	Ma et al. [30]
Western North Pacific	16–22 May 2004	30		Morphology & 16S	Ma et al. [30]
North Equatorial Current in the Western North Pacific	13 May-6 Jul 2004	37	5*	Morphology & 16S	Miyazaki et al. [31]
	27 May-16 Jul 2005				
	26 Jun-05 Sep 2006				
	3 Aug–15 Sep 2007				
	21 May–14 Jul 2008				
	14 Apr–3 Jun 2009				
*Gnathophis heterognathos*					
East China Sea	27 Nov-7 Dec 2000	29	4*^,^ **	Morphology	Kurogi et al. [6]
Northeastern Japan	17–23 Oct 2003	27	6*	Morphology & 16S	Kimura et al. [3]
Off Jeju Is.	24–29 Aug 2014	33	7**	Morphology & 12S	Ji et al. [32]
For eggs and larvae					
East China sea	29 Jun– 13 Jul 2009	25	8*	COI	Lin et al. [18]

^†^ Sampling methods: 1, Bongo net (diameter 0.6 m; mesh 333 μm, towing time ~10 min); 2, Bongo net and Isaacs Kidd midwater trawls (IKMT) (features on net is not indicated); 3, RN80 net (features on net is not indicated); 4, IKMT (mouth opening 8.7 m^2^; mesh 1.0 or 0.5 mm); 5, IKMT and ORI Big-Fish plankton net (respectively mouth opening 8.7 and 7.1 m^2^; mesh, 0.5 mm); 6, IKMT (mouth opening 8.7 m^2^; mesh 0.5 mm); 7, ORI (Ocean Research Institute) net (diameter 2 m; mesh 500 μm); 8, RMI (using a round-mouth ichthyoplankton) net (diameter 1.3 m; mesh 1.0 mm).

* Oblique towing.

** horizontal towing.

^‡^ Species identification is based on molecular genetic markers (12S rRNA, 16S rRNA, COI, and cytochrome b sequences of mitochondrial DNA) and/or morphology.

## Results

### Molecular genetic identification of the eggs

Sixteen of 33,432 eggs were identified as belonging to four species: *Ophisurus macrorhynchos*, *Echelus uropterus*, *Ariosoma majus*, and *Gnathophis heterognathos*. Primary analysis of COI sequences from 32,753 eggs or 16S rRNA sequences from 679 eggs designated 16 eggs as Anguilliformes. Then the species of these 16 eggs were identified by reference to the sequences of two other molecular genetic markers of either 12S and 16S rRNA or 12S rRNA and COI. Geographical information about adults of the genus *Ophisurus* was also used to identify the eggs.

**Fig 2:**
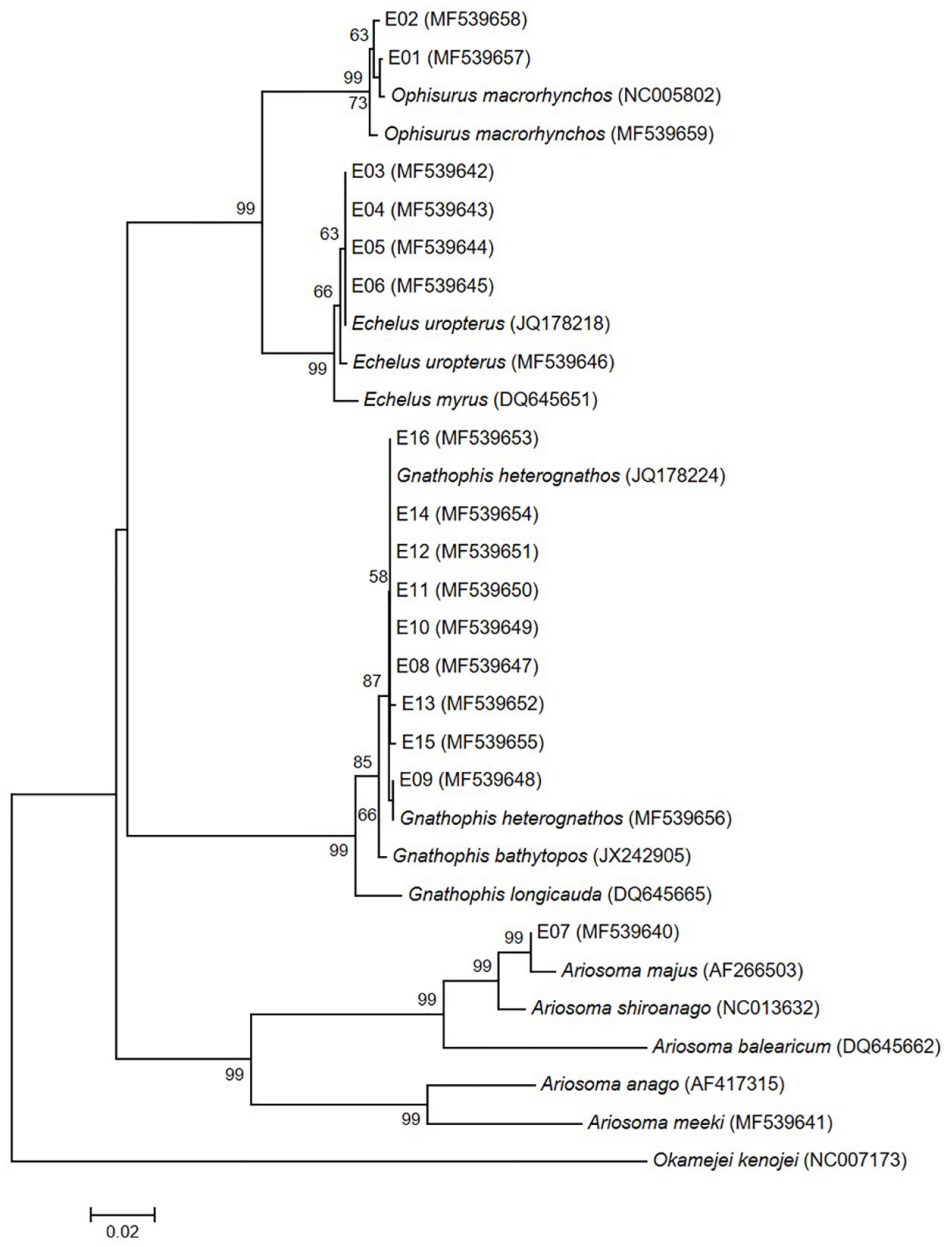
Neighbor-joining tree of 12S rRNA sequences for the eggs and four genera of eels (*Ophisurus*, *Echelus*, *Ariosoma*, and *Gnathohpis*). E01-E16 indicates each egg. Others are sequences from four genera of eels and from *Okamejei kenojei* (as outgroup species). Numbers next to the branches are bootstrap values (>50%).

**Fig 3:**
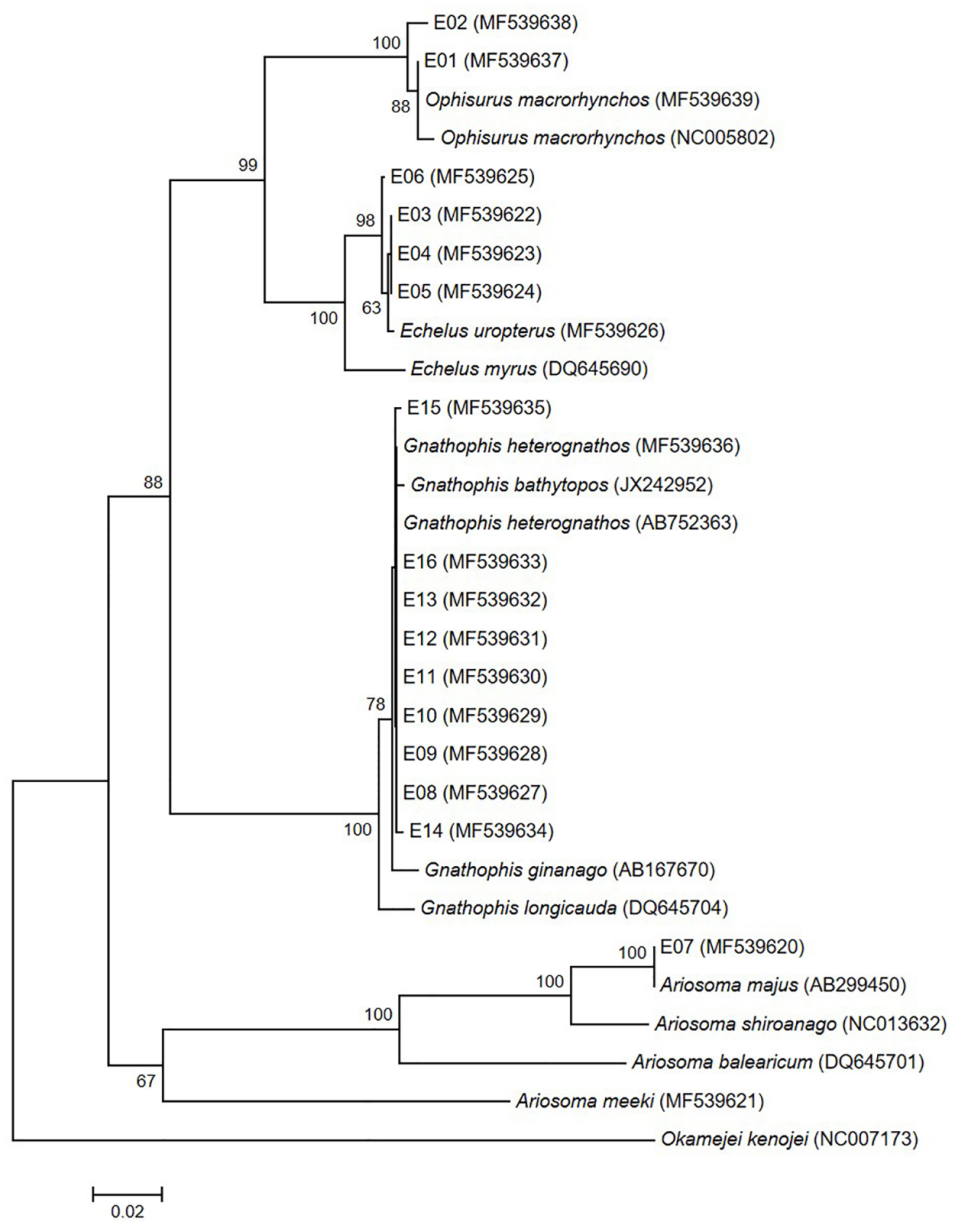
Neighbor-joining tree of 16S rRNA sequences for the eggs and four genera of eels (*Ophisurus*, *Echelus*, *Ariosoma*, and *Gnathohpis*). E01-E16 indicates each egg. Others are sequences from four genera of eels and from *Okamejei kenojei* (as outgroup species). Numbers next to the branches are bootstrap values (>50%).

**Fig 4:**
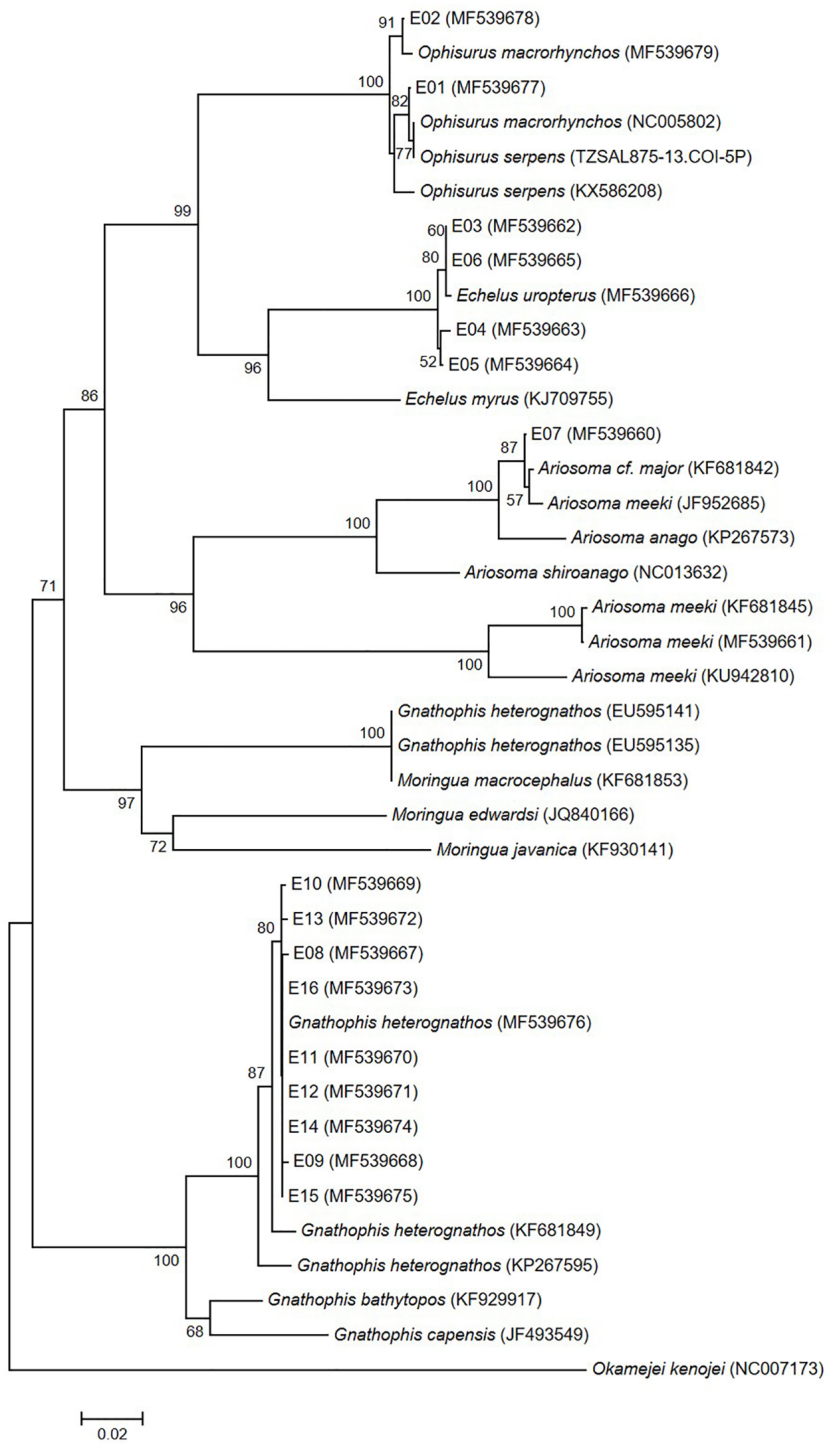
Neighbor-joining tree of COI sequences for the eggs and five genera of eels (*Ophisurus*, *Echelus*, *Ariosoma*, *Gnathohpis*, and *Moringua*). E01-E16 indicates each egg. Others are sequences from five genera of eels and from *Okamejei kenojei* (as outgroup species). Numbers next to the branches are bootstrap values (>50%).

Two eggs (E01 and E02) were identified as those of *Ophisurus macrorhynchos* (Bleeker 1852) using both 12S and 16S rRNA sequence data and the geographical distribution of adult eels. The genetic distances between the eggs and *O*. *macrorhynchos* 12S and 16S rRNA sequences were very small (0.002–0.007 for 12S rRNA; 0–0.013 for 16S rRNA) (Figs 2 and 3; S1 and S2 Tables). According to the COI sequences, the eggs (MF539677 and MF539678) formed a clade with *O*. *macrorhynchos* (NC005802 and MF539679) and *O*. *serpens* (TZSAL875-13.COI-5P and KX586208) (Fig 4). The genetic distances within this clade (0.000–0.018) were too small to distinguish the two species (S3 Table). Fortunately, adults and leptocephali of *O*. *macrorhynchos* were found in the East China Sea [7,34,35]. The eggs were thus determined to be *O*. *macrorhynchos* based on adult geographical information.

Four eggs (E03–E06) were those of *Echelus uropterus* (Temminck & Schlegel 1846). The COI sequence data were used to distinguish among species of the genus *Echelus*. The four eggs and *E*. *uropterus* formed a single clade in neighbor-joining trees of all three DNA regions tested (Figs 2–4). The genetic distances were very small: 0–0.007 (COI) (S3 Table) and 0–0.004 (12S and 16S rRNA) (S1 and S2 Tables). Interspecific genetic distances for two of the tested DNA regions (12S and 16S rRNA) were also small between this clade and *E*. *myrus* (0.009–0.030) (S1 and S2 Tables). COI had the greatest interspecific divergence (0.101–0.103) (S3 Table).

One egg (E07) was identified as belonging to *Ariosoma majus* (Asano 1958) based on a comparison of genetic distances of the 12S and 16S rRNA sequences with those of the genus *Ariosoma*. The COI sequence from a local adult specimen of *A*. *meeki* (MF539661) was used to correct uncertain sequences. The genetic distance based on 16S rRNA between the egg (MF539620) and *A*. *major* (AB299450) was zero (Fig 3 and S2 Table) and that from 12S rRNA was very small (0.007; Fig 2 and S1 Table). Interspecific genetic distances based on 16S rRNA between the egg and the genus *Ariosoma* (0.047–0.233) were larger than those obtained using 12S rRNA (≥0.018). In the COI neighbor-joining tree, the egg (MF539660) formed a clade with both *A*. *meeki* (JF952685) and *A*. cf. *major* (KF681842) (genetic distances, 0.004–0.005) (Fig 4 and S3 Table). This *A*. *meeki* (JF952685) was distinct from other *A*. *meeki* sequences, including one from a local adult (MF539661). Based on the COI sequence of the local specimen (MF539661) and the 12S and 16S rRNA genetic distances within the genus *Ariosoma*, the sequences labeled *A*. *meeki* (JF952685) and *A*. cf. *major* (KF681842) were likely both *A*. *majus*.

Nine eggs (E08–E16) were identified as those of *Gnathophis heterognathos* (Bleeker 1858) by reference to the COI sequence of a local adult specimen (MF539676). The COI genetic distances between the eggs and the *G*. *heterognathos* specimens (MF539676, KF681849, and KP267595) (0.000–0.022) were smaller than those between the eggs and both *G*. *bathytopos* (KF929917) and *G*. *capensis* (JF493549) (0.053–0.081) (S3 Table). Sequences from *G*. *heterognathos* (EU595135 and EU595141) made up a distinct clade (Fig 4), which diverged sharply (0.196–0.206) from the *G*. *heterognathos* clade containing the eggs. The two divergent sequences of *G*. *heterognathos* (EU595135 and EU595141) matched a *Moringua macrocephalus* sequence (KF681853). In the 12S and 16S rRNA neighbor-joining trees, the eggs formed a clade with the genus *Gnathophis*. The genetic distances within this clade were too small (0.000–0.028 for 12S rRNA; 0–0.024 for 16S rRNA) to clearly delineate the two species, but suggested that the eggs belonged to the genus *Gnathophis* (Figs 2 and 3; S1 and S2 Tables).

### Egg morphology

**Fig 5:**
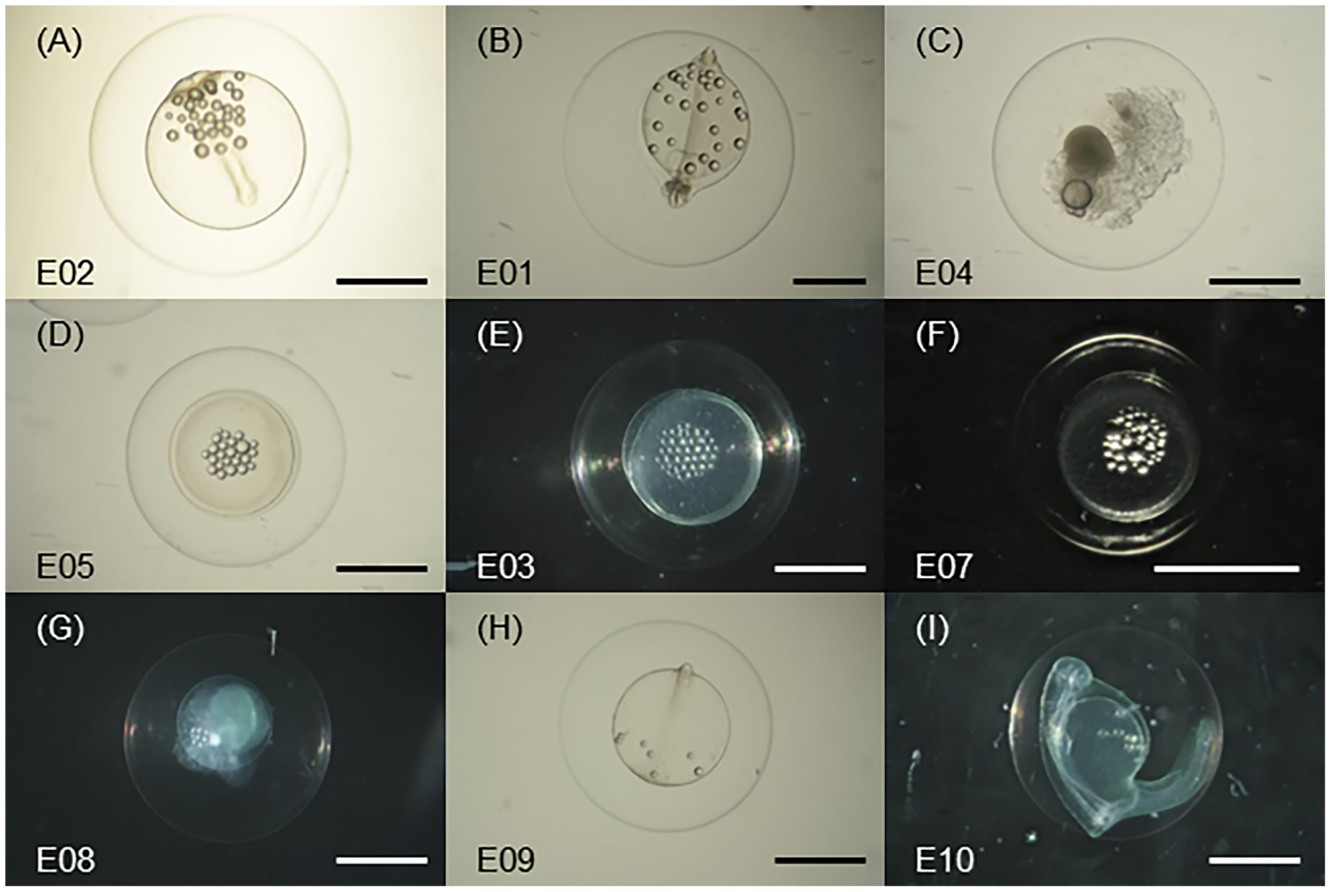
The egg morphologies of four species of Ophichthidae and Congridae. (A, B) *Ophisurus macrorhynchos*. (C, D, E) *Echelus uropterus*. (F) *Ariosoma majus*. (G, H, I) *Gnathophis heterognathos*. The letters in the lower left regions of the photographs are the specimen identification numbers. Scale bars, 1 mm.

The eggs of all four species of Ophichthidae and Congridae were round and pelagic and were sampled in the early or middle developmental stages (Fig 5). Egg diameters ranged from 1.5 to 3.2 mm. The smallest egg was that of *Ariosoma majus* (Fig 5F) and the largest was that of *Ophisurus macrorhynchos* (Fig 5B). All eggs had wide perivitelline spaces. The numbers of oil globules ranged from 3 to 35; the number and location of oil globules differed by developmental stage, with lower numbers during early developmental stages (Fig 5C and 5G). The oil globules first appeared clustered (Fig 5D, 5E and 5F) and then spread across the yolk (Fig 5A, 5B, 5H and 5I). The developmental stages of these eggs were after perivitelline space formation for the *A*. *majus* egg; prior to embryonic body formation to the late stage for *Gnathophis heterognathos* eggs; optic vesicle formation stage for *O*. *macrorhynchos* eggs; and early stage to after perivitelline space formation for *Echelus uropterus* eggs.

### Distributions of eggs and leptocephali

**Fig 6:**
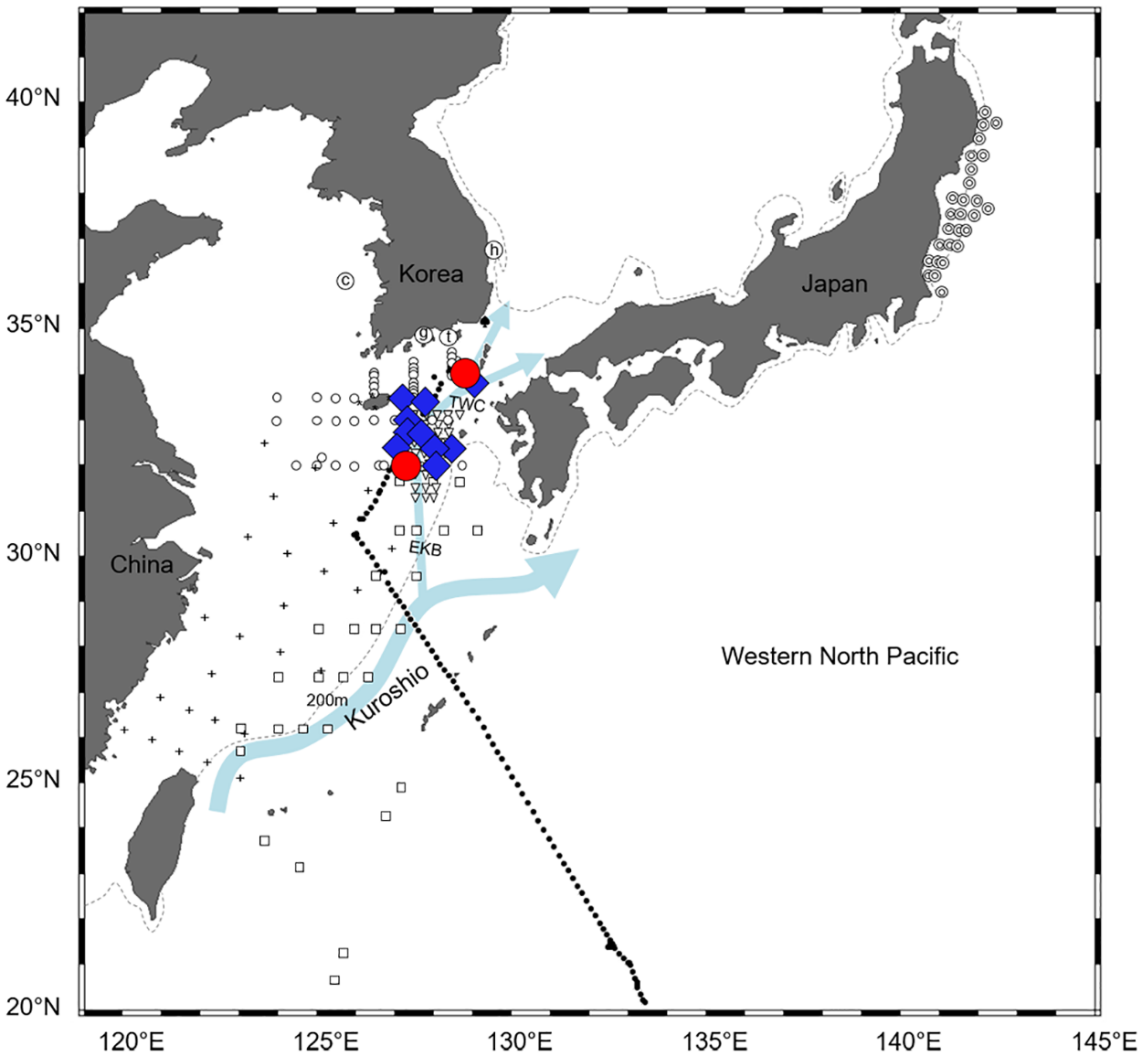
The distribution of eggs and leptocephali of *Ophisurus macrorhynchos*. Eggs of *O*. *macrorhynchos* collected in this study (August 2016) are shown with red circles. Leptocephali of *O*. *macrorhynchos* (August 2013 and 2014, 9.8–44.5 mm TL) [7] are shown with blue diamonds. The other markings indicate surveys for fish eggs and/or larvae, but with no catches for these of *O*. *macrorhynchos*. Table 1 describes for the markings: circled letter c, g, h, and t, open circle, and black dot. Table 5 describes for the markings: bullseye, [3]; square, [6,30]; cross, [18]; spade, [21]; asterisk, [29]; crossed circle, [30]; up-pointing triangle, [31]; and down-pointing triangle, [32]. Arrows indicate ocean currents: The Kuroshio, Eastern Kuroshio Branch (EKB), and Tsushima Warm Current (TWC) [36]. Dashed line means 200m depth contour.

**Fig 7:**
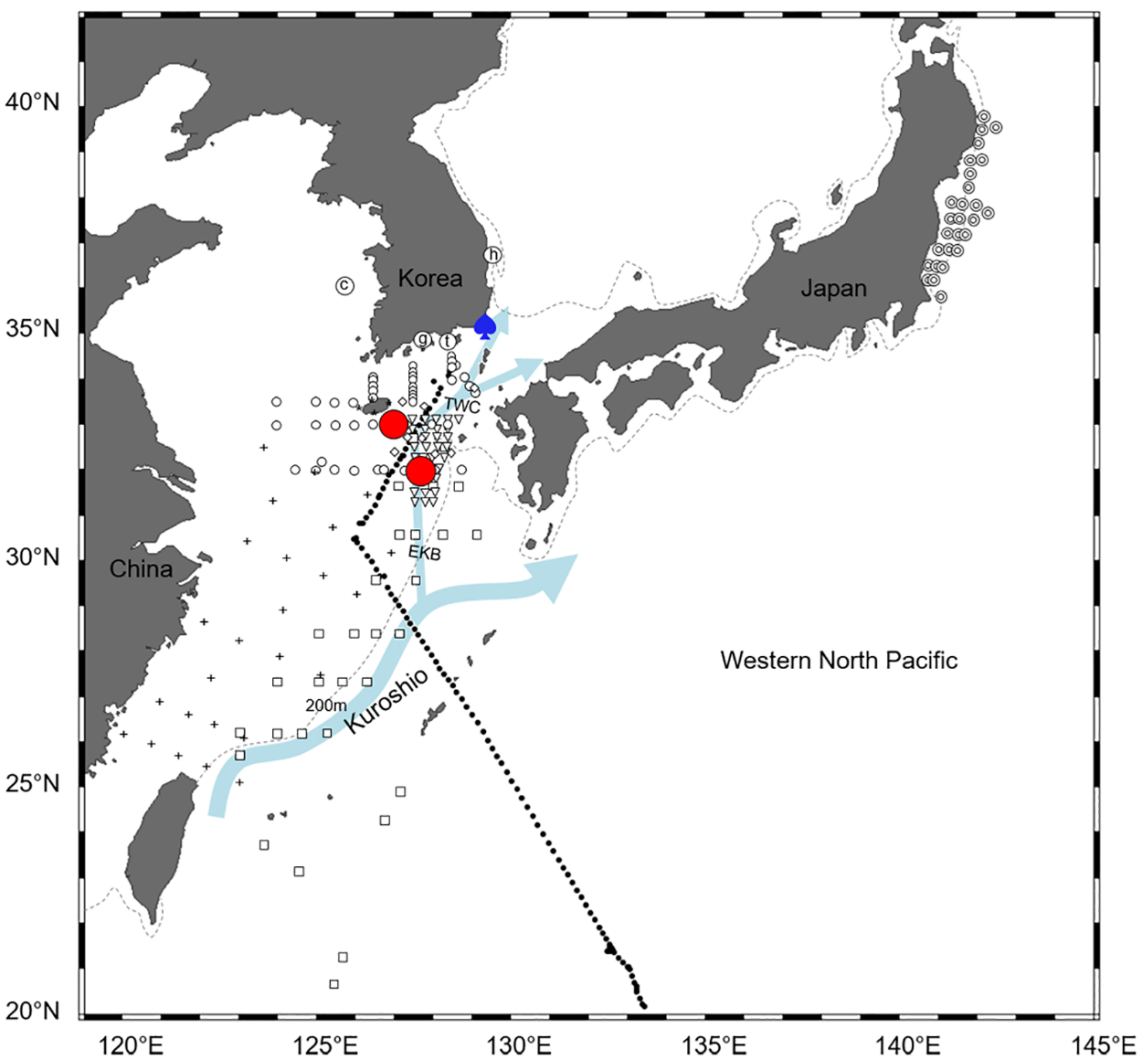
The distribution of eggs and leptocephali of *Echelus uropterus*. Eggs of *E*. *uropterus* collected in this study (August 2016) are shown with red circles. Leptocephali of *E*. *uropterus* (July-October 2010, 14.6–68 mm TL) [21] are shown with blue spade. The other markings indicate surveys for fish eggs and/or larvae, but with no catches for these of *E*. *uropterus*. Table 1 describes for the markings: circled letter c, g, h, and t, open circle, and black dot. Table 5 describes for the markings: bullseye, [3]; square, [6,30]; diamond, [7]; cross, [18]; asterisk, [29], crossed circle, [30]; up-pointing triangle, [31]; and down-pointing triangle, [32]. Arrows indicate ocean currents: The Kuroshio, Eastern Kuroshio Branch (EKB), and Tsushima Warm Current (TWC) [36]. Dashed line means 200m depth contour.

**Fig 8:**
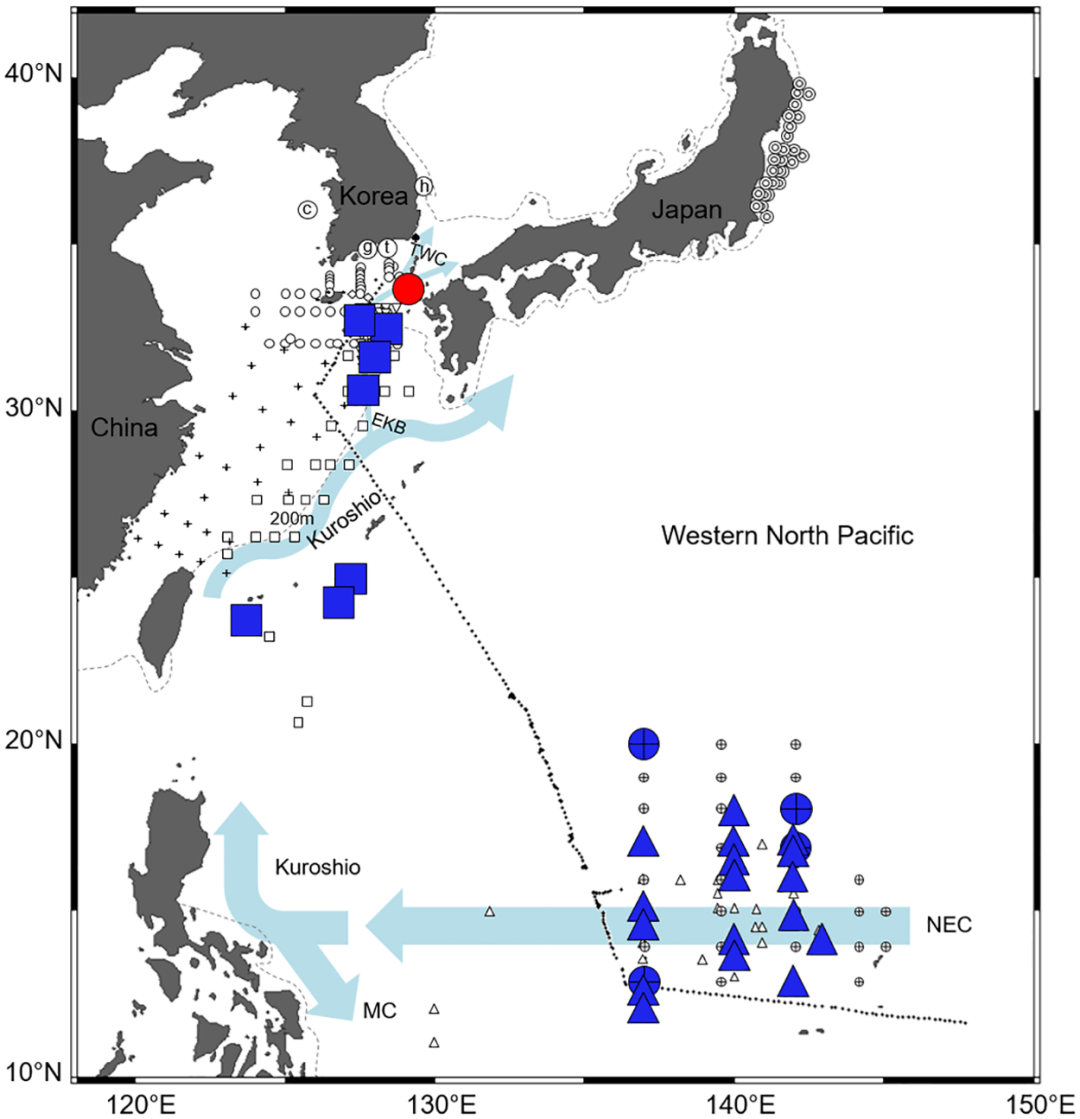
The distribution of egg and leptocephali of *Ariosoma majus*. Egg of *A*. *majus* collected in this study (August 2016) is shown with red circle. Leptocephali of *A*. *majus* are shown with blue squares (November to December 2000, 40–148 mm in TL, [30]), blue crossed circles (May 2004, 105–151 mm in TL, [30]), and blue up-pointing triangles (April to September during 2004–2009, 88.0–317.5 mm in TL, [31]). The other markings indicate surveys for fish eggs and/or larvae, but with no catches for these of *A*. *majus*. Table 1 describes for the markings: circled letter c, g, h, and t, open circle, and black dot. Table 5 describes for the markings: bullseye, [3]; square, [6]; diamond, [7]; cross, [18]; spade, [21]; asterisk, [29]; and down-pointing triangle, [32]. Arrows indicate ocean currents: The Kuroshio, Eastern Kuroshio Branch (EKB), and Tsushima Warm Current (TWC) in East China Sea [36] and the Kuroshio Current and Mindanao Current (MC) branched from the North Equatorial Current (NEC) [37]. Dashed line means 200m depth contour.

**Fig 9:**
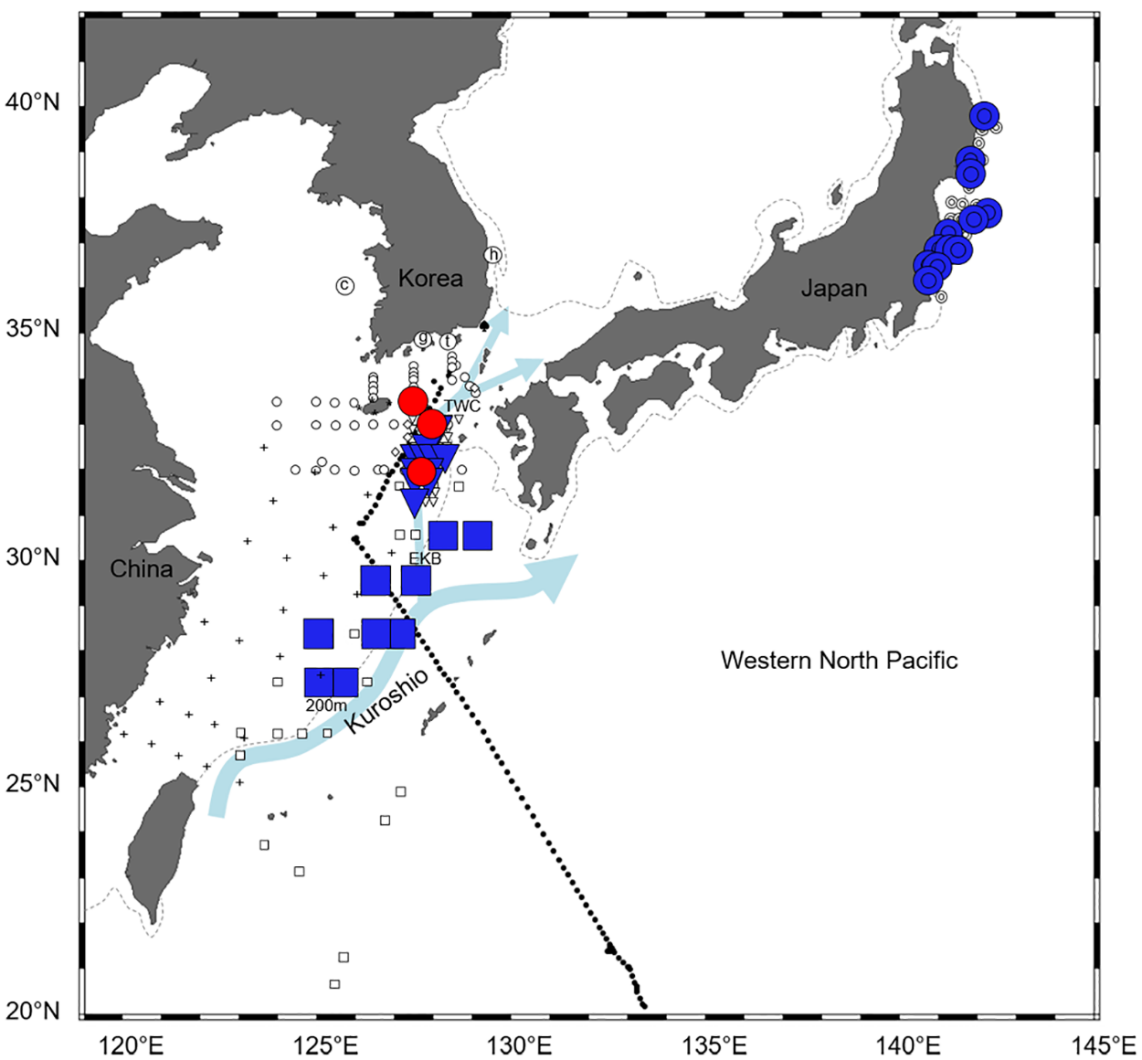
The distribution of eggs and leptocephali of *Gnathohpis heterognathos*. Eggs of *G*. *heterognathos* collected in this study (August 2016) are shown with red circles. Leptocephali of *G*. *heterognathos* are shown with blue bullseyes (October 2003, 3.8–59.8 mm TL, [3]), blue squares (November to December 2000, 6.8–88.8 mm TL, [6]), and blue down-pointing triangles (August 2014, 15.8–32.3 mm TL, [32]) The other markings indicate surveys for fish eggs and/or larvae, but with no catches for these of *G*. *heterognathos*. Table 1 describes for the markings: circled letter c, g, h, and t, open circle, and black dot. Table 5 describes for the markings: diamond, [7]; cross, [18]; spade, [21]; asterisk, [29]; square and crossed circle, [30]; and up-pointing triangle, [31]. Arrows indicate ocean currents: The Kuroshio, Eastern Kuroshio Branch (EKB), and Tsushima Warm Current (TWC) [36]. Dashed line means 200m depth contour.

Eggs of all four species of Ophichthidae and Congridae were found over the continental shelf of the northern East China Sea in August 2016. Sea surface temperatures ranged from 24.2°C to 31.4°C during the survey and was relatively high (30.3–31.1°C) where the eggs were collected. Leptocephali of the same species were also found near our study area (Figs 6–9). The distribution ranges varied greatly by taxon. The leptocephali of *Ophisurus macrorhynchos* (August, 9.8–44.5 mm total length [TL]) [7] and *Echelus uropterus* (July-October, 14.6–68 mm TL) [21] were found only where the eggs were detected (Figs 6 and 7). Leptocephali of the Congridae have been collected in several widely distributed areas, including where the eggs were detected. Leptocephali of *Ariosoma majus* were found in some places of the East China Sea (November to December, 40–148 mm TL), the western North Pacific (May, 105–151 mm TL) [30], and the North Equatorial Current (April to September, 88.0–317.5 mm TL) [31] (Fig 8). Leptocephali of *Gnathophis heterognathos* were found near the southeastern Jeju Island (August, 15.8–32.3 mm TL) [32], the East China Sea (December, 6.8–88.8 mm TL) [6], and off the northeastern coast of Japan (October, 3.8–59.8 mm TL) [3] (Fig 9).

## Discussion

### Identification of the eggs using multi-genetic markers

Identification of fish eggs to the species level is essential for locating spawning areas [13]. Most studies have used only a single molecular genetic marker to this purpose [8,17,29]. When using genetic markers, three issues must be considered: comparative sequences may be lacking, intra- and interspecific divergence may vary with the genetic markers used, and publicly available sequences sometimes represent misidentified specimens. We confirmed the species of all eggs using three molecular genetic markers, geographical distributional data, and sequences from local adult specimens. Sequences from both local fish specimens and public databases are necessary to identify eggs to the species level, partly because the sequences needed to compare interspecific genetic variation may be unavailable. For *Echelus uropterus*, only 12S rRNA sequences were publicly available to estimate interspecific divergence within the genus *Echelus*. The 12S rRNA genetic distances between *E*. *uropterus* (JQ178218 and MF539646) and *E*. *myrus* (DQ645651) (0.004–0.013) were small (S1 Table). Thus, we obtained 16S rRNA (MF539626) and COI sequences (MF539666) from a local adult specimen *E*. *uropterus* (Figs 3 and 4; S2 and S3 Tables). The COI sequence optimally distinguished this species from others.

In addition, some public sequences are derived from misidentified specimens in the genera *Gnathophis* and *Ariosoma*. In terms of neighbor-joining tree based on COI, one *G*. *heterognathos* clade (EU595135 and EU595141) diverged notably from the clade including *G*. *heterognathos* (KF681849, KP267595, and MF539676) and eggs E08–E16. The genetic distances between the clades ranged from 0.196 to 0.206 (Fig 4 and S3 Table). The latter clade contained a sequence from an adult *G*. *heterognathos* specimen (MF539676) collected near our study area. Thus, we determined that the sequences of EU595135 and EU595141, which made up a distinct clade, were attributable to misidentification. These two sequences matched a *Moringua macrocephalus* sequence (KF681853) (Fig 4). In the genus *Ariosoma*, one COI clade contained the egg E07 (MF539660), *A*. *meeki* (JF952685), and a species *incertae sedis* labeled *A*. *cf*. *major* (KF681842). The *A*. *cf*. *major* (KF681842) and *A*. *meeki* (JF952685) sequences would be those of *A*. *majus* based on both their divergence from the clade containing the *A*. *meeki* (MF539661) and 16S rRNA sequence of egg E07 (MF539620) which was identical to that of *A*. *majus* (AB299450). The sequence MF539661 was obtained from a local adult specimen.

Due to these issues, we also used geographical information to identify the species associated with eggs. It was impossible to explore interspecific genetic divergence within *Ophisurus* using 12S rRNA and 16S rRNA sequences, because these sequences were available only for *O*. *macrorhynchos*, one of two species in the genus [28]. As the COI interspecific divergence between *O*. *serpens* (TZSAL875-13.COI-5P and KX586208) and *O*. *macrorhynchos* (NC005802 and MF539679) was small (0.000–0.018), these two species may be the same species. However, adults and leptocephali of only *O*. *macrorhynchos* are found near our study area [7,34,35]. Such geographical information was critical for identifying eggs E01 and E02 to *O*. *macrorhynchos*.

### Distribution of eggs and leptocephali

Four species of eggs (Figs 6–9), *Ophisurus macrorhynchos*, *Echelus uropterus*, *Ariosoma majus*, and *Gnathophis heterognathos*, were first genetically discovered over the continental shelf of the northern East China Sea in August 2016. These eggs were in either the early or middle developmental stages (Fig 5). Although it is difficult to use morphological characteristics of eggs to identify their species, collections of eggs provide useful information for locating spawning areas, since areas near the locations of eggs in early developmental stages have a high probability of being spawning areas. Considering the high seawater temperatures (30.3–31.1°C) where the eggs were found, spawning had occurred 1–2 days prior to sampling of eggs [38]. The place where these species spawn could be located as far away as 50 km in the downstream direction of the Eastern Kuroshio Branch (mean speed, 30 cm/s; [36]) which passes where the eggs were found.

The location of a spawning area can also be estimated based on the growth rate and pelagic duration of leptocephali. The average growth rate of leptocephali, *Anguilla japonica*, *Saurenchelys stylura*, and *Dysomma sp*., is 0.56 mm/day [39–41]. Applying this growth rate to the minimum leptocephali three species, *Ophisurus macrorhynchos*, *Echelus uropterus*, and *Gnathophis heterognathos* (6.8 to 15.8 mm; [6,7,21,32]), their pelagic durations before capture might be about 12–28 days. Considering the mean speed of the Eastern Kuroshio branch [36], these leptocephali could have drifted a few hundred kilometers from the site where they were spawned. In addition to transport by oceanic currents, tidal currents and vertical migration of leptocephali must be considered when attempting to locate spawning areas [42]. These results indicate that locating spawning sites using eggs is simpler and more accurate than using leptocephali.

Leptocephali of *Ophisurus macrorhynchos* and *Echelus uropterus* were found near the area where their eggs were found, and leptocephali of *Gnathophis heterognathos* and *Ariosoma majus* were distributed over a wide area including where their eggs appeared. Leptocephali of *G*. *heterognathos* have also been observed off the northeastern coast of Japan (October, 3.8–59.8 mm TL) [3] (Fig 9). Applying the daily growth rate mentioned above to the smallest leptocephali of *G*. *heterognathos* (3.8 mm TL), their age was estimated to be 4 days post-hatching, and thus they would have hatched from eggs spawned off the northeastern coast of Japan. Leptocephali of *A*. *majus* were found in the East China Sea, the open sea of the Ryukyu Island Arc (40–148 mm TL) [30], and near the North Equatorial Current (105–151 mm TL, [30]; 88–317.5 mm TL; [31]) (Fig 8). It would be difficult to identify spawning areas using this leptocephalus distribution information.

The region where the eggs and leptocephali of the four species were found indicates that the northern East China Sea could be spawning areas of these species. The collection of the eggs of all four species during August, is consistent with the summer to fall spawning seasons of marine eels in this region that has been reported previously through leptocephali [43]. Future research to genetically identify eggs and leptocephali will be able to provide useful information to understand the spawning locations and larval distributions of marine eels in this region.

## Supporting information

S1 Fig Sampling stations for fish eggs in the northern East China Sea and the southern Korean Peninsula during four times surveys from June 2015 to August 2016. (PDF)

S1 Table Genetic 12S rRNA distances between eggs and four genera (*Ophisurus*, *Echelus*, *Ariosoma*, and G*nathophis*) of the Anguilliformes. (PDF)

S2 Table Genetic 16S rRNA distances between eggs and four genera (*Ophisurus*, *Echelus*, *Ariosoma*, and *Gnathophis*) of the Anguilliformes. (PDF)

S3 Table Genetic cytochrome c oxidase subunit 1 (COI) distances between eggs and five genera (*Ophisurus*, *Echelus*, *Ariosoma*, *Gnathophis*, and *Moringua*) of the Anguilliformes. (PDF)
