# Supplementary material for: Genetic identification of eggs from four species of Ophichthidae and Congridae (Anguilliformes) in the northern East China Sea
Source: PLoS One. 2018 Apr 5;13(4):e0195382. doi: 10.1371/journal.pone.0195382 (PMC5886565; doi:10.1371/journal.pone.0195382)
Supplement: S1 Fig — (PDF) [file pone.0195382.s001.pdf]

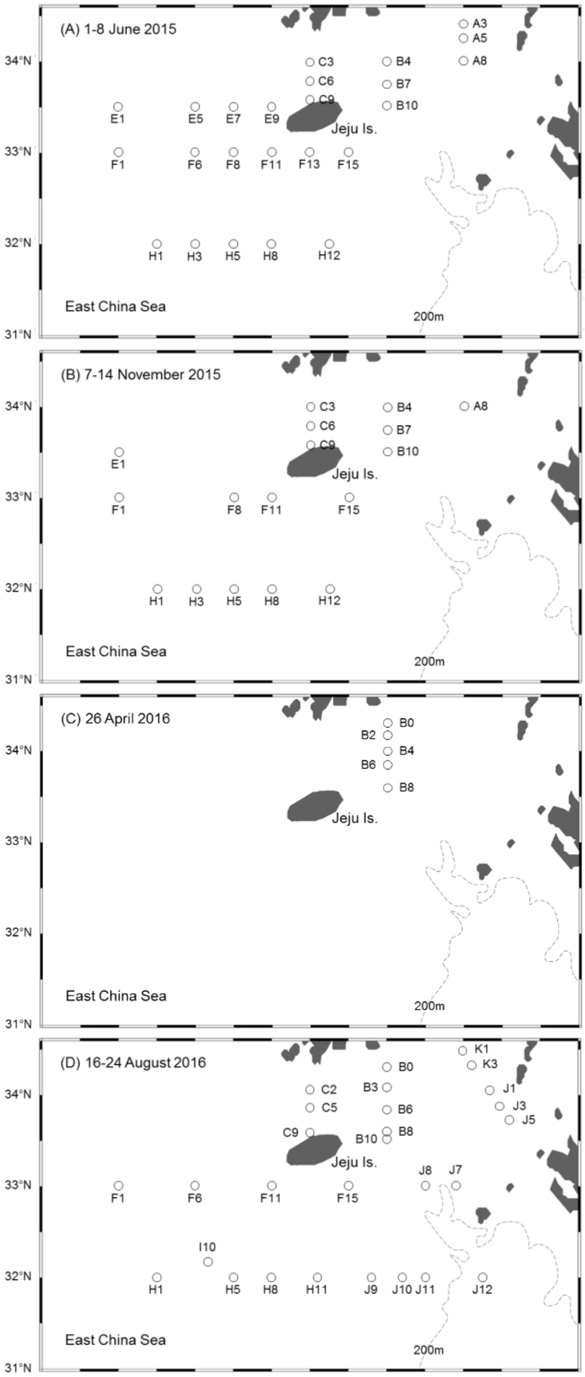

S1 Fig. Sampling stations for fish eggs in the northern East China Sea and the southern Korean Peninsula during four times surveys from June 2015 to August 2016. Dashed line means 200m depth contour.
