# Supplementary material for: Genetic identification of eggs from four species of Ophichthidae and Congridae (Anguilliformes) in the northern East China Sea
Source: PLoS One. 2018 Apr 5;13(4):e0195382. doi: 10.1371/journal.pone.0195382 (PMC5886565; doi:10.1371/journal.pone.0195382)
Supplement: S1 Table — (PDF) [file pone.0195382.s002.pdf]

S1 Table. Genetic 12S rRNA distances between eggs and four genera (*Ophisurus, Echelus*, *Ariosoma,* and *Gnathophis*) of the Anguilliformes.

	E01	E02	1	2	E03	E04	E05	E06	3	4	5	E07	6	7	8	9	10	E08	E09	E10	E11	E12	E13	E14	E15	E16	11	12	13	14	15
E01				*Ophisurus*																											
E02	0.004																														
1	0.002	0.005					`																								
2	0.005	0.005	0.007																												
E03	0.067	0.063	0.069	0.067							*Echelus*																				
E04	0.067	0.063	0.069	0.067	0																										
E05	0.067	0.063	0.069	0.067	0	0																									
E06	0.067	0.063	0.069	0.067	0	0	0																								
3	0.067	0.063	0.069	0.067	0	0	0	0																							
4	0.069	0.065	0.071	0.063	0.004	0.004	0.004	0.004	0.004																						
5	0.065	0.065	0.067	0.065	0.009	0.009	0.009	0.009	0.009	0.013																					
E07	0.214	0.211	0.213	0.212	0.199	0.199	0.199	0.199	0.199	0.199	0.204						*Ariosoma*														
6	0.219	0.216	0.219	0.217	0.199	0.199	0.199	0.199	0.199	0.199	0.204	0.018																			
7	0.221	0.218	0.220	0.218	0.206	0.206	0.206	0.206	0.206	0.206	0.211	0.007	0.026																		
8	0.267	0.264	0.267	0.265	0.265	0.265	0.265	0.265	0.265	0.265	0.273	0.091	0.091	0.099																	
9	0.206	0.202	0.206	0.206	0.184	0.184	0.184	0.184	0.184	0.189	0.189	0.186	0.194	0.195	0.217																
10	0.225	0.223	0.225	0.227	0.202	0.202	0.202	0.202	0.202	0.207	0.200	0.185	0.188	0.194	0.221	0.083															
E08	0.166	0.166	0.165	0.164	0.152	0.152	0.152	0.152	0.152	0.152	0.157	0.216	0.211	0.226	0.237	0.232	0.246													*Gnathophis*	
E09	0.163	0.163	0.163	0.161	0.150	0.150	0.150	0.150	0.150	0.150	0.155	0.214	0.209	0.223	0.234	0.229	0.243	0.002													
E10	0.166	0.166	0.165	0.164	0.152	0.152	0.152	0.152	0.152	0.152	0.157	0.216	0.211	0.226	0.237	0.232	0.246	0	0.002												
E11	0.166	0.166	0.165	0.164	0.152	0.152	0.152	0.152	0.152	0.152	0.157	0.216	0.211	0.226	0.237	0.232	0.246	0	0.002	0											
E12	0.166	0.166	0.165	0.164	0.152	0.152	0.152	0.152	0.152	0.152	0.157	0.216	0.211	0.226	0.237	0.232	0.246	0	0.002	0	0										
E13	0.163	0.163	0.163	0.161	0.154	0.154	0.154	0.154	0.154	0.155	0.159	0.216	0.211	0.226	0.239	0.229	0.243	0.002	0.004	0.002	0.002	0.002									
E14	0.166	0.166	0.165	0.164	0.152	0.152	0.152	0.152	0.152	0.152	0.157	0.216	0.211	0.226	0.237	0.232	0.246	0	0.002	0	0	0	0.002								
E15	0.168	0.168	0.168	0.166	0.150	0.150	0.150	0.150	0.150	0.150	0.155	0.216	0.211	0.226	0.237	0.229	0.243	0.002	0.004	0.002	0.002	0.002	0.004	0.002							
E16	0.166	0.166	0.165	0.164	0.152	0.152	0.152	0.152	0.152	0.152	0.157	0.216	0.211	0.226	0.237	0.232	0.246	0	0.002	0	0	0	0.002	0	0.002						
11	0.163	0.163	0.163	0.161	0.150	0.150	0.150	0.150	0.150	0.150	0.155	0.214	0.209	0.223	0.234	0.229	0.243	0.002	0	0.002	0.002	0.002	0.004	0.002	0.004	0.002					
12	0.166	0.166	0.165	0.164	0.152	0.152	0.152	0.152	0.152	0.152	0.157	0.216	0.211	0.226	0.237	0.232	0.246	0	0.002	0	0	0	0.002	0	0.002	0	0.002				
13	0.163	0.163	0.163	0.161	0.154	0.154	0.154	0.154	0.154	0.155	0.155	0.222	0.216	0.231	0.239	0.234	0.246	0.005	0.007	0.005	0.005	0.005	0.007	0.005	0.007	0.005	0.007	0.005			
14	0.167	0.168	0.167	0.168	0.154	0.154	0.154	0.154	0.154	0.156	0.151	0.230	0.224	0.239	0.248	0.239	0.248	0.026	0.028	0.026	0.026	0.026	0.028	0.026	0.028	0.026	0.028	0.026	0.020		
15	0.313	0.310	0.316	0.305	0.322	0.322	0.322	0.322	0.322	0.317	0.325	0.383	0.390	0.392	0.382	0.367	0.379	0.320	0.320	0.320	0.320	0.320	0.323	0.320	0.323	0.320	0.320	0.320	0.329	0.323	

E01-E16: eggs collected in this study. E01 (GenBank accession number, MF539657); E02 (MF539658); E03 (MF539642); E04 (MF539643); E05 (MF539644); E06 (MF539645); E07 (MF539640); E08 (MF539647); E09 (MF539648); E10 (MF539649); E11 (MF539650); E12 (MF539651); E13 (MF539652); E14 (MF539654); E15 (MF539655); E16 (MF539653); 1, *Ophisurus macrorhynchos* (NC005802); 2, *Ophisurus macrorhynchos* (MF539659); 3, *Echelus uropterus* (JQ178218); 4, *Echelus uropterus* (MF539646); 5, *Echelus myrus* (DQ645651); 6, *Ariosoma shiroanago* (NC013632); 7, *Ariosoma majus* (AF266503); 8, *Ariosoma balearicum* (DQ645662); 9, *Ariosoma anago* (AF417315); 10, *Ariosoma meeki* (MF539641); 11, *Gnathophis heterognathos* (MF539656); 12, *Gnathophis heterognathos* (JQ178224); 13, *Gnathophis bathytopos* (JX242905); 14, *Gnathophis longicauda* (DQ645665); 15, *Okamejei kenojei* (NC007173).
