# Supplementary material for: Genetic identification of eggs from four species of Ophichthidae and Congridae (Anguilliformes) in the northern East China Sea
Source: PLoS One. 2018 Apr 5;13(4):e0195382. doi: 10.1371/journal.pone.0195382 (PMC5886565; doi:10.1371/journal.pone.0195382)
Supplement: S2 Table — (PDF) [file pone.0195382.s003.pdf]

S2 Table. Genetic 16S rRNA distances between eggs and four genera (*Ophisurus, Echelus*, *Ariosoma,* and *Gnathophis*) of the Anguilliformes.

	E01	E02	1	2	E03	E04	E05	E06	3	4	E07	5	6	7	8	E08	E09	E10	E11	E12	E13	E16	E14	E15	9	10	11	12	13	14
E01			*Ophisurus*																											
E02	0.009																													
1	0	0.009																												
2	0.004	0.013	0.004																											
E03	0.081	0.084	0.081	0.086						*Echelus*																				
E04	0.081	0.084	0.081	0.086	0																									
E05	0.081	0.084	0.081	0.086	0	0																								
E06	0.079	0.081	0.079	0.084	0.002	0.002	0.002																							
3	0.084	0.086	0.084	0.088	0.002	0.002	0.002	0.004																						
4	0.086	0.088	0.086	0.091	0.028	0.028	0.028	0.030	0.030																					
E07	0.232	0.238	0.232	0.232	0.223	0.223	0.223	0.223	0.226	0.231					*Ariosoma*															
5	0.232	0.238	0.232	0.232	0.223	0.223	0.223	0.223	0.226	0.231	0																			
6	0.214	0.214	0.214	0.220	0.193	0.193	0.193	0.190	0.196	0.192	0.233	0.233																		
7	0.232	0.232	0.232	0.232	0.223	0.223	0.223	0.223	0.226	0.229	0.047	0.047	0.233																	
8	0.229	0.229	0.229	0.229	0.234	0.234	0.234	0.234	0.237	0.237	0.136	0.136	0.247	0.144																
E08	0.138	0.140	0.138	0.143	0.130	0.130	0.130	0.128	0.130	0.135	0.253	0.253	0.201	0.251	0.233														*Gnathophis*	
E09	0.138	0.140	0.138	0.143	0.130	0.130	0.130	0.128	0.130	0.135	0.253	0.253	0.201	0.251	0.233	0														
E10	0.138	0.140	0.138	0.143	0.130	0.130	0.130	0.128	0.130	0.135	0.253	0.253	0.201	0.251	0.233	0	0													
E11	0.138	0.140	0.138	0.143	0.130	0.130	0.130	0.128	0.130	0.135	0.253	0.253	0.201	0.251	0.233	0	0	0												
E12	0.138	0.140	0.138	0.143	0.130	0.130	0.130	0.128	0.130	0.135	0.253	0.253	0.201	0.251	0.233	0	0	0	0											
E13	0.138	0.140	0.138	0.143	0.130	0.130	0.130	0.128	0.130	0.135	0.253	0.253	0.201	0.251	0.233	0	0	0	0	0										
E16	0.138	0.140	0.138	0.143	0.130	0.130	0.130	0.128	0.130	0.135	0.253	0.253	0.201	0.251	0.233	0	0	0	0	0	0									
E14	0.140	0.143	0.140	0.146	0.128	0.128	0.128	0.125	0.128	0.133	0.257	0.257	0.204	0.254	0.236	0.002	0.002	0.002	0.002	0.002	0.002	0.002								
E15	0.138	0.140	0.138	0.143	0.133	0.133	0.133	0.130	0.133	0.138	0.253	0.253	0.198	0.25	0.234	0.002	0.002	0.002	0.002	0.002	0.002	0.002	0.004							
9	0.138	0.140	0.138	0.143	0.130	0.130	0.130	0.128	0.130	0.135	0.253	0.253	0.201	0.251	0.233	0	0	0	0	0	0	0	0.002	0.002						
10	0.138	0.140	0.138	0.143	0.130	0.130	0.130	0.128	0.130	0.135	0.253	0.253	0.201	0.251	0.233	0	0	0	0	0	0	0	0.002	0.002	0					
11	0.140	0.143	0.140	0.146	0.133	0.133	0.133	0.130	0.133	0.138	0.257	0.257	0.198	0.254	0.236	0.002	0.002	0.002	0.002	0.002	0.002	0.002	0.004	0.004	0.002	0.002				
12	0.148	0.151	0.148	0.148	0.144	0.144	0.144	0.141	0.144	0.141	0.247	0.247	0.210	0.244	0.230	0.015	0.015	0.015	0.015	0.015	0.015	0.015	0.017	0.015	0.015	0.015	0.017			
13	0.148	0.151	0.148	0.154	0.141	0.141	0.141	0.138	0.141	0.146	0.260	0.260	0.201	0.251	0.236	0.009	0.009	0.009	0.009	0.009	0.009	0.009	0.011	0.011	0.009	0.009	0.011	0.024		
14	0.309	0.323	0.309	0.316	0.306	0.306	0.306	0.306	0.306	0.303	0.352	0.352	0.349	0.359	0.353	0.301	0.301	0.301	0.301	0.301	0.301	0.301	0.298	0.301	0.301	0.301	0.305	0.298	0.291	

E01-E16: eggs collected in this study. E01 (GenBank accession number, MF539637); E02 (MF539638); E03 (MF539622); E04 (MF539623); E05 (MF539624); E06 (MF539625); E07 (MF539620); E08 (MF539627); E09 (MF539628); E10 (MF539629); E11 (MF539630); E12 (MF539631); E13 (MF539632); E14 (MF539634); E15 (MF539635); E16 (MF539633); 1, *Ophisurus macrorhynchos* (MF539639); 2, *Ophisurus macrorhynchos* (NC005802); 3, *Echelus uropterus* (MF539626); 4, *Echelus myrus* (DQ645690); 5, *Ariosoma majus* (AB299450); 6, *Ariosoma meeki* (MF539621); 7, *Ariosoma shiroanago* (NC013632); 8, *Ariosoma balearicum* (DQ645701); 9, *Gnathophis heterognathos* (AB752363); 10, *Gnathophis heterognathos* (MF539636); 11, *Gnathophis bathytopos* (JX242952); 12, *Gnathophis longicauda* (DQ645704); 13, *Gnathophis ginanago* (AB167670); 14, *Okamejei kenojei* (NC007173).
