# Supplementary material for: Genetic identification of eggs from four species of Ophichthidae and Congridae (Anguilliformes) in the northern East China Sea
Source: PLoS One. 2018 Apr 5;13(4):e0195382. doi: 10.1371/journal.pone.0195382 (PMC5886565; doi:10.1371/journal.pone.0195382)
Supplement: S3 Table — (PDF) [file pone.0195382.s004.pdf]

S3 Table. Genetic cytochrome c oxidase subunit 1 (COI) distances between eggs and five genera (*Ophisurus*, *Echelus*, *Ariosoma*, *Gnathophis*, and *Moringua*) of the Anguilliformes.

	E01	E02	1	2	3	4	E03	E04	E05	E06	5	6	E07	7	8	9	10	11	12	13	E08	E09	E10	E11	E12	E13	E14	E15	E16	14	15	16	17	18	19	20	21	22	23	24
E01						*Ophisurus*																																		
E02	0.009																																							
1	0.002	0.007																																						
2	0.013	0.004	0.011																																					
3	0.002	0.007	0	0.011																																				
4	0.013	0.015	0.011	0.018	0.011																																			
E03	0.154	0.154	0.156	0.156	0.156	0.154						*Echelus*																												
E04	0.157	0.157	0.159	0.159	0.159	0.156	0.005																																	
E05	0.157	0.157	0.159	0.159	0.159	0.156	0.005	0.004																																
E06	0.154	0.154	0.156	0.156	0.156	0.154	0	0.005	0.005																															
5	0.154	0.154	0.156	0.156	0.156	0.154	0.002	0.007	0.007	0.002																														
6	0.130	0.130	0.128	0.133	0.128	0.128	0.101	0.101	0.101	0.101	0.103																													
E07	0.246	0.248	0.243	0.251	0.243	0.243	0.217	0.214	0.212	0.217	0.217	0.225																								*Ariosoma*				
7	0.248	0.251	0.246	0.254	0.246	0.246	0.219	0.217	0.214	0.219	0.219	0.227	0.004																											
8	0.251	0.254	0.248	0.257	0.248	0.249	0.222	0.219	0.217	0.222	0.222	0.230	0.005	0.005																										
9	0.256	0.259	0.253	0.262	0.253	0.254	0.229	0.227	0.224	0.229	0.229	0.240	0.034	0.034	0.028																									
10	0.217	0.219	0.219	0.222	0.219	0.217	0.233	0.230	0.233	0.233	0.233	0.220	0.080	0.084	0.082	0.084																								
11	0.262	0.257	0.262	0.254	0.262	0.249	0.262	0.260	0.257	0.262	0.265	0.262	0.222	0.225	0.228	0.233	0.235																							
12	0.262	0.257	0.262	0.254	0.262	0.249	0.265	0.262	0.260	0.265	0.268	0.259	0.220	0.222	0.225	0.230	0.233	0.002																						
13	0.257	0.251	0.257	0.249	0.257	0.243	0.267	0.265	0.262	0.267	0.270	0.248	0.209	0.212	0.215	0.220	0.228	0.057	0.055																					
E08	0.209	0.206	0.211	0.211	0.211	0.214	0.215	0.218	0.212	0.215	0.217	0.205	0.245	0.248	0.253	0.261	0.206	0.275	0.272	0.259																				
E09	0.204	0.201	0.206	0.206	0.206	0.209	0.220	0.223	0.218	0.220	0.222	0.200	0.245	0.248	0.253	0.266	0.206	0.275	0.272	0.259	0.004																			
E10	0.209	0.206	0.211	0.211	0.211	0.214	0.220	0.223	0.218	0.220	0.222	0.205	0.245	0.248	0.253	0.266	0.206	0.270	0.267	0.259	0.004	0.004																		
E11	0.206	0.204	0.209	0.209	0.209	0.211	0.217	0.220	0.215	0.217	0.220	0.203	0.242	0.245	0.250	0.264	0.204	0.272	0.270	0.256	0.002	0.002	0.002																	
E12	0.206	0.204	0.209	0.209	0.209	0.211	0.217	0.220	0.215	0.217	0.220	0.203	0.242	0.245	0.250	0.264	0.204	0.272	0.270	0.256	0.002	0.002	0.002	0																
E13	0.204	0.201	0.206	0.206	0.206	0.209	0.215	0.218	0.212	0.215	0.217	0.200	0.245	0.248	0.253	0.266	0.201	0.270	0.267	0.254	0.004	0.004	0.004	0.002	0.002															
E14	0.206	0.204	0.209	0.209	0.209	0.211	0.217	0.220	0.215	0.217	0.220	0.203	0.242	0.245	0.250	0.264	0.204	0.272	0.270	0.256	0.002	0.002	0.002	0	0	0.002														
E15	0.206	0.204	0.209	0.209	0.209	0.211	0.217	0.220	0.215	0.217	0.220	0.203	0.242	0.245	0.250	0.264	0.204	0.272	0.270	0.256	0.002	0.002	0.002	0	0	0.002	0													
E16	0.206	0.204	0.209	0.209	0.209	0.211	0.217	0.220	0.215	0.217	0.220	0.203	0.242	0.245	0.250	0.264	0.204	0.272	0.270	0.256	0.002	0.002	0.002	0	0	0.002	0	0												
14	0.206	0.204	0.209	0.209	0.209	0.211	0.217	0.220	0.215	0.217	0.220	0.203	0.242	0.245	0.250	0.264	0.204	0.272	0.270	0.256	0.002	0.002	0.002	0	0	0.002	0	0	0											
15	0.207	0.204	0.209	0.204	0.209	0.209	0.213	0.215	0.210	0.213	0.215	0.205	0.252	0.255	0.261	0.277	0.216	0.264	0.262	0.259	0.013	0.013	0.013	0.011	0.011	0.013	0.011	0.011	0.011	0.011										
16	0.206	0.204	0.209	0.204	0.209	0.206	0.217	0.220	0.215	0.217	0.220	0.200	0.242	0.244	0.250	0.263	0.209	0.270	0.267	0.254	0.020	0.020	0.020	0.018	0.018	0.020	0.018	0.018	0.018	0.018	0.022									
17	0.209	0.206	0.211	0.201	0.211	0.208	0.227	0.227	0.222	0.227	0.230	0.200	0.252	0.255	0.260	0.268	0.224	0.267	0.264	0.262	0.057	0.057	0.053	0.055	0.055	0.053	0.055	0.055	0.055	0.055	0.063	0.063								
18	0.206	0.201	0.203	0.196	0.203	0.201	0.219	0.222	0.217	0.219	0.222	0.192	0.277	0.280	0.285	0.291	0.245	0.267	0.264	0.259	0.081	0.081	0.077	0.079	0.079	0.077	0.079	0.079	0.079	0.079	0.083	0.081	0.055							
19	0.244	0.247	0.247	0.250	0.247	0.250	0.238	0.238	0.236	0.238	0.236	0.233	0.249	0.252	0.252	0.257	0.260	0.247	0.244	0.255	0.199	0.201	0.196	0.199	0.199	0.196	0.199	0.199	0.199	0.199	0.206	0.199	0.190	0.206						
20	0.244	0.247	0.247	0.250	0.247	0.250	0.238	0.238	0.236	0.238	0.236	0.233	0.249	0.252	0.252	0.257	0.260	0.247	0.244	0.255	0.199	0.201	0.196	0.199	0.199	0.196	0.199	0.199	0.199	0.199	0.206	0.199	0.190	0.206	0					
21	0.244	0.247	0.247	0.250	0.247	0.250	0.238	0.238	0.236	0.238	0.236	0.233	0.249	0.252	0.252	0.257	0.260	0.247	0.244	0.255	0.199	0.201	0.196	0.199	0.199	0.196	0.199	0.199	0.199	0.199	0.206	0.199	0.190	0.206	0	0			*Moringua*	
22	0.208	0.208	0.208	0.210	0.208	0.202	0.217	0.217	0.219	0.217	0.217	0.202	0.262	0.262	0.265	0.260	0.249	0.255	0.258	0.255	0.199	0.197	0.197	0.199	0.199	0.197	0.199	0.199	0.199	0.199	0.202	0.199	0.199	0.209	0.158	0.158	0.158			
23	0.216	0.211	0.216	0.214	0.216	0.216	0.231	0.226	0.228	0.231	0.233	0.238	0.275	0.275	0.278	0.267	0.251	0.297	0.300	0.304	0.214	0.217	0.211	0.214	0.214	0.217	0.214	0.214	0.214	0.214	0.219	0.212	0.209	0.206	0.179	0.179	0.179	0.153		
24	0.300	0.300	0.303	0.300	0.303	0.297	0.360	0.358	0.361	0.360	0.360	0.308	0.400	0.400	0.400	0.393	0.355	0.352	0.352	0.346	0.275	0.281	0.275	0.278	0.278	0.281	0.278	0.278	0.278	0.278	0.278	0.278	0.284	0.281	0.316	0.316	0.316	0.316	0.321	

E01-E16: eggs collected in this study. E01 (GenBank accession number, MF539677); E02 (MF539678); E03 (MF539662); E04 (MF539663); E05 (MF539664); E06 (MF539665); E07 (MF539660); E08 (MF539667); E09 (MF539668); E10 (MF539669); E11 (MF539670); E12 (MF539671); E13 (MF539672); E14 (MF539674); E15 (MF539675); E16 (MF539673); 1, *Ophisurus macrorhynchos* (NC005802); 2, *Ophisurus macrorhynchos* (MF539679); 3, *Ophisurus serpens* (TZSAL875-13.COI-5P); 4, *Ophisurus serpens* (KX586208); 5, *Echelus uropterus* (MF539666); 6, *Echelus myrus* (KJ709755); 7, *Ariosoma cf. major* (KF681842); 8, *Ariosoma meeki* (JF952685); 9, *Ariosoma anago* (KP267573); 10, *Ariosoma shiroanago* (NC013632); 11, *Ariosoma meeki* (KF681845); 12, *Ariosoma meeki* (MF539661); 13, *Ariosoma meeki* (KU942810); 14, *Gnathophis heterognathos* (MF539676); 15, *Gnathophis heterognathos* (KF681849); 16, *Gnathophis heterognathos* (KP267595); 17, *Gnathophis bathytopos* (KF929917); 18, *Gnathophis capensis* (JF493549); 19, *Gnathophis heterognathos* (EU595141); 20, *Gnathophis heterognathos* (EU595135); 21, *Moringua microcephalus* (KF681853); 22, *Moringua edwardsi* (JQ840166); 23, *Moringua javanica* (KF930141); 24, *Okamejei kenojei* (NC007173).
